# Supplementary material for: Soybean Seed Enrichment with Cobalt and Molybdenum as an Alternative to Conventional Seed Treatment
Source: Plants (Basel). 2023 Mar 3;12(5):1164. doi: 10.3390/plants12051164 (PMC10007214; doi:10.3390/plants12051164)
Supplement: Supplementary file 1 [file plants-12-01164-s001.zip › plants-2204069-supplementary.pdf]

plants

MDPI

Table S1. Aboveground and seeds dry matter (DM) of soybean crops under Co and Mo application via foliar and via soil in greenhouse conditions.

Dose (g ha^{-1})	Foliar	Soil	Foliar	Soil
Co/Mo	Aerial Part DM (g per Pot)		Seeds DM (g per Pot)	
0/0	6.5 A		5.7 A	
0/800	4.7 A	5.3 A	5.6 A	5.9 A
10/800	4.6 A	5.4 A	5.4 A	4.8 A
20/800	4.4 A	5.5 A	4.8 A	5.5 A
30/800	6.0 A	4.8 A	6.0 A	4.3 A
Average	4.9 a	5.3 a	5.4 a	5.1 a
CV (%)	16.0		28.7	

FTS—early cultivar; M640—conventional cultivar. Averages followed by the same capital letter, vertically, do not differ from the control by the Dunnett test ($p < 0.05$). Averages followed by the same lower-case letter, in the horizontal line, do not differ statistically by the Tukey test ($p < 0.05$).

Table S2. Pearson's correlation between Co and Mo contents in seeds and aerial part of soybean parent plant, and in soil, with seed quality and yield components of soybean parent plants treated with foliar application with Co and Mo, under field conditions.

**Attributes**		**CI**	**FI**	**NBI**	**GR**	**AAGR**	**GI**	**UI**	**VI**	**Yield**
	Seed	0.11	−0.02	0.06	0.34	0.59	0.37	0.25	0.38	0.02
Co	Aerial Part	−0.04	0.03	−0.02	0.20	0.56	0.31	0.37	0.34	0.10
	Soil	−0.01	−0.42	0.36	0.03	−0.03	0.06	0.23	0.11	−0.01
	Seed	−0.17	−0.20	0.09	−0.06	0.42	0.24	0.36	0.27	−0.21
Mo	Aerial Part	−0.15	−0.04	−0.05	−0.14	0.45	−0.004	0.13	0.004	−0.18
	Soil	0.02	−0.18	0.16	−0.05	−0.14	0.17	0.09	0.19	−0.29

CI—chlorophyl index; FI—flavonoid index; NBI—nitrogen balance index; GR—germination rate; AAGR—accelerated aging germination rate; GI—growth index; UI—development uniformity index; VI—vigor index. Cells filled with red, orange, and yellow show that there was a significant effect, respectively, at 0.05, 0.01, and 0.001 by the *t*-test. Without filling, there was no significance by the *t*-test ($p > 0.05$).

Table S3. Pearson's correlation between Co and Mo contents in seeds and aerial part of soybean parent plant, and in soil, with nutrient contents in seeds, and aerial part of soybean parent plants treated with foliar application with Co and Mo, under field conditions.

Nutrients		N	P	K	Ca	Mg	S	B	Fe	Mn	Zn	Cu
						**Seed**						
Co	Seed	0.007	−0.36	0.003	−0.61	−0.65	−0.58	−0.34	−0.53	−0.73	−0.42	−0.61
	Aerial Part	−0.04	−0.32	−0.13	−0.67	−0.66	−0.57	−0.31	−0.55	−0.73	−0.40	−0.58
	Soil	−0.06	−0.21	0.01	0.10	0.18	0.03	0.28	0.03	0.21	0.14	0.11
Mo	Seed	−0.06	−0.41	0.08	−0.41	−0.24	−0.22	−0.07	−0.70	−0.45	−0.46	−0.25
	Aerial Part	−0.21	−0.27	−0.01	−0.42	−0.20	−0.21	−0.26	−0.60	−0.57	−0.36	−0.20
	Soil	0.23	−0.08	−0.10	0.05	0.03	−0.002	0.22	−0.07	0.07	−0.05	0.30
						**Aerial Part**						
Co	Seed	0.25	−0.27	0.16	−0.29	−0.14	−0.15	−0.27	0.04	−0.32	0.29	−0.07
	Aerial Part	0.23	−0.35	0.14	−0.30	−0.19	−0.09	−0.30	0.08	−0.45	0.29	0.02
	Soil	−0.07	−0.37	0.16	−0.08	−0.17	−0.43	0.21	0.13	0.03	0.05	0.20
Mo	Seed	0.09	−0.20	0.22	−0.19	−0.07	−0.05	0.27	0.04	−0.25	0.23	0.19
	Aerial Part	0.02	0.001	0.4	−0.33	−0.27	0.33	0.21	0.24	−0.36	0.41	0.47
	Soil	−0.02	−0.15	−0.25	0.04	0.22	−0.12	0.03	−0.36	0.06	−0.08	−0.003

Cells filled with red, orange, and yellow show that there was a significant effect, respectively, at 0.05, 0.01, and 0.001 by the *t*-test. Without filling, there was no significance by the *t*-test ($p > 0.05$).
